# On terahertz pulsed broadband Gauss-Bessel beam free-space propagation

**DOI:** 10.1038/s41598-018-19830-z

**Published:** 2018-01-23

**Authors:** Maksim S. Kulya, Varvara A. Semenova, Victor G. Bespalov, Nikolay V. Petrov

**Affiliations:** 1 ITMO University, International Institute “Photonics and Optoinformatics”, Kadetskaya line 3, 199034 Russia; 2 ITMO University, Laboratory of Digital and Display Holography, St. Petersburg, Kadetskaya line 3, 199034 Russia

## Abstract

Terahertz pulse time-domain holography is the ultimate technique allowing the evaluating a propagation of pulse broadband terahertz wavefronts and analyze their spatial, temporal and spectral evolution. We have numerically analyzed pulsed broadband terahertz Gauss-Bessel beam’s both spatio-temporal and spatio-spectral evolution in the non-paraxial approach. We have characterized two-dimensional spatio-temporal beam behavior and demonstrated all stages of pulse reshaping during the propagation, including X-shape pulse forming. The reshaping is also illustrated by the energy transfer dynamics, where the pulse energy flows from leading edge to trailing edge. This behavior illustrates strong spatio-temporal coupling effect when spatio-temporal distribution of Bessel beam’s wavefront depends on propagation distance. The spatio-temporal and spatio-spectral profiles for different spectral components clearly illustrate the model where the Bessel beam’s wavefront at the exit from the axicon can be divided into radial segments for which the wave vectors intersect. Phase velocity via propagation distance is estimated and compared with existing experimantal results. Results of the phase velocity calculation depend strongly on distance increment value, thus demonstrating superluminal or subluminal behavior.

## Introduction

Bessel beam is a theoretical model of non-diffracting light beam with an infinite number of rings that can cover an infinite distance and require an infinite amount of power. Although true non-diffractive Bessel beam can not be created in practice, several approximations can be made. The beams obtained in such approximations are called quasi-Bessel beams and exhibit low or no diffraction over a limited propagation distance. One of the most efficient ways for quasi-Bessel beams generation is Gaussian beam focusing by an axicon or conical lens. Gauss-Bessel beam formation by the axicon occurs due to a linear phase-delay in a transverse coordinate for an incident light field. A Gauss-Bessel light beam is generated at the axicon exit with an amplitude distribution in the radial cross-section described by the square of the zeroth-order Bessel function. Incident radiation can be both continuous wave or pulsed wave.

At present, quasi-Bessel light beams attract wide attention from scientists due to a number of unique properties^1^: high intensity in the near-axis region over a limited propagation distance, small diffraction divergence of the central maximum in comparison with traditional Gaussian beams, and reconstruction properties. Quasi-Bessel beams are also actual in case of non-linear light-matter iteractions: these beams enhance ionization and laser-induced plasma effects^2^. Quasi-Bessel beams are widely used in optical coherence tomography^3^, control of micro- and nanoparticles^4^, high-precision microprocessing^5^, detection of rotating objects^6^ and three-dimensional imaging^7^.

The concept of non-diffractive beams can be theoretically implemented for any range of electromagnetic radiation. Recently, studies of terahertz (THz) Bessel beams properties began, and Bessel beams of narrow and broadband THz radiation have already found their application in several fields. For example, usage of Bessel-like beams in THz imaging results in significantly improved depth of focus^8^. Another actively developing application based on THz radiation is THz communications, where the Bessel beam’s potential for wireless communications was studied^9^. THz communications are a promising technology to satisfy the increasing requirements on a capacity and speed of data transmission in wireless systems^10–12^. THz communication systems were developed on the basis of continuous and narrow-band THz signal sources. However, the possibilities of using broadband THz signals, maximizing the available bandwidth of THz frequencies, are already being discussed^13^. Broadband THz systems applicability for communications is restricted by the attenuation and diffraction during the propagation in the atmosphere. This problem is especially important for communications. One possible solution which may be addressed to this problem is the application of broadband THz Bessel beams for data signal transfer. Spreading immunity of quasi-Bessel beams seems even more useful for the case of pulsed radiation, when we should control both spatial and temporal light behavior. However, to apply non-dispersive or, which is more exact, limited dispesive properties of pulsed Bessel beams for practical purposes, it is necessary to investigate the formation and propagation features of pulsed Bessel beams of broadband THz radiation.

First of all, pulsed broadband Bessel beams (also called “X-shaped waves”) are different from monochromatic Bessel beams as they contain multiple frequencies. Therefore, such beams are localized (i.e. maintain their shape) at a single frequency, but become dispersive for multiple frequencies, because the phase velocity of each frequency component is different. Thus, X-shaped waves are non-dispersive only in isotropic-homogeneous media. Secondly, propagation behavior of X-shaped waves is much more complex, since superluminal effects occur in their speed evolution. The term X-shaped waves appeared because they represent X shaped intensity distribution over time and radius and result from the interference of few-cycle wavepackets from ultrashort pulse light sources. Some papers mentioned that due to the “scissors effect”, the point of intersection of two or more crossing wave fronts should not obey the restrictions imposed by the principle of relativity^14,15^. As a result, the interference maximum at the apex of the axicon can propagate at a velocity exceeding the speed of light in vacuum. The observation of this effect^16^ led to series of discussions^14,15,17,18^. Although it is obvious that the energy velocity is always subluminal, one can observe an effect that beam propagating along the optical axis has superluminal group and phase velocity value. In general, these superluminal effects can be explained both by a specific geometry of the wave front during the passage of THz radiation through the axicon, and by the large anomalous dispersion of the propagation media^19^. However, axicon generated Bessel beam’s faster-than-light propagation is not related to the absorption or dispersion anomaly but arises due to the interference between the plane wave components of the beam in free space^20^.

Superluminal effects for evanescent waves have been demonstrated in tunneling experiments^21^. This effect can be revealed only over short distances due to the evanescent field properties. Then there were attempts performed to extend this effect over larger distance: Mugnai *et al*.^16^. demonstrated such a possibility in the propagation of localized microwaves over the distance of some tens of wavelengths. Experimental evidence of localized light waves in a centimeter range was given in^22^, demonstrating a practical way of obtaining X-shaped waves. These waves were theoretically predicted as Bessel beams in the 1980’s^23^, and then they were investigated in connection with their superluminal behavior^24^. Recently, measuring of a spatio-temporal field structure of Bessel X-shaped beams has been performed for ultrashort pulses in paper^25^.

In the THz frequency range Bessel beams evolution deserves additional attention because of ultimate case of a pulsed radiation consisting of a few electric field oscillations. Since the appearance of the coherent detection technique, which allows direct measurement of THz amplitude field in temporal domain, there have been several attempts to estimate experimentally superluminal effect of pulsed THz Bessel beam propagation. For example, in the work of Lloyd *et al*.^26^ faster-than-light behavior was observed, but only in the optical beam axis. Pulse reshaping was also determined for paraxial case, but it does not illustrate full spatio-temporal pulse behavior during propagation, especiaclly in presence of spatio-temporal coupling (STC), analyzed for ultrashort pulsed THz radiation^27,28^. Moreover, the sampling distance step in experimental measurements of superluminal effect in^26^ for THz waveforms exceeded almost an order of value of the size occupied by the beam in space.

Since the key issue is the experimental realization of X-waves and the monitoring of their evolution, there is ongoing effort in developing methods and tools for such studies. THz imaging is a possible solution allowing mathematical modeling and simulation of THz field passed through the object^29–31^. However, most of the imaging techniques do not provide a full description of spatio-temporal evolution of THz field, and can only form images of simple binary amplitude objects. Holographic approach outperforms imaging based on time-domain spectroscopy, which is more powerfull for the chemical sciences^32^, or tomographic^33^ techniques in acquisition time. THz holography^34,35^ provides also better spatial resolution, and needs less computational powers for numerical reconstruction than tomography. THz pulse time-domain holography (THz PTDH)^36^ as a specific case of the holography method applied for pulsed THz radiation provides information about temporal and complex spectral characteristics of the wave field. Field propagation through an arbitrary amplitude-phase object allows its relief and optical characteristics reconstruction. THz PTDH developed for measuring the amplitude-phase characteristics of a field passed through an object provides wide possibilities for analyzing a dynamics of complex wavefront^37^. This technique is a powerful tool that allows extrapolation of the THz field behavior in spatial regions located at different distances from the plane where the measurements are provided.

Research of pulsed THz Gauss-Bessel beam’s full spatio-temporal and spatio-spectral evolution via propagation distance is actual and has not performed yet by our knowledge. The purpose of the research is to study the propagation dynamics of the THz pulse two-dimensional profile during the passage through the phase axicon in the spatio-temporal and spatio-spectral representations, as well as the estimation of the phase velocity behavior for THz Gauss-Bessel beam in non-paraxial approach and comparison with existing experimental results.

The paper is organized as follows. Section “Results” considers the spatio-temporal and the spatio-spectral evolution of pulsed broadband THz Gauss-Bessel beam. Spatio temporal coupling and pulse reshaping are demonstrated in time-domain. In the spectral domain the broadband evolution is demonstrated as well as dynamics for the individual frequency components, thus illustrating that the wavefront after the axicon consists of radially symmetric segments which propagate at certain angles to the optical axis. Broadband longitudinal spectral evolution is additionally presented. The section “Phase velocity estimation” provides the description of pecularities of superluminal effect calculation, estimating the contribution of distance increment along *z*-axis.

## Results

### Spatio-temporal evolution

The electric field of any ultrashort laser pulse often fails to be separated purely into a temporal and spatial factors. These effect known as spatio-temporal coupling (STC) is observed for instance in review^28^. STC is important in many studies connected with wavefront propagation. THz pulse consisting of only several oscillations of electric field is a special case of pulsed electromagnetic radiation which should be also investigated according to this STC effect. Moreover, Gauss-Bessel beam’s evolution represents a non-paraxial case of wavefront propagation due to the fact that radially symmetric segments of the wavefront propagate along optical *z*-axis at some angle after the axicon. In this case temporal (or spectral) and spatial (or angular) properties of THz ultrashort pulse are interdependent.

**Figure 1:**
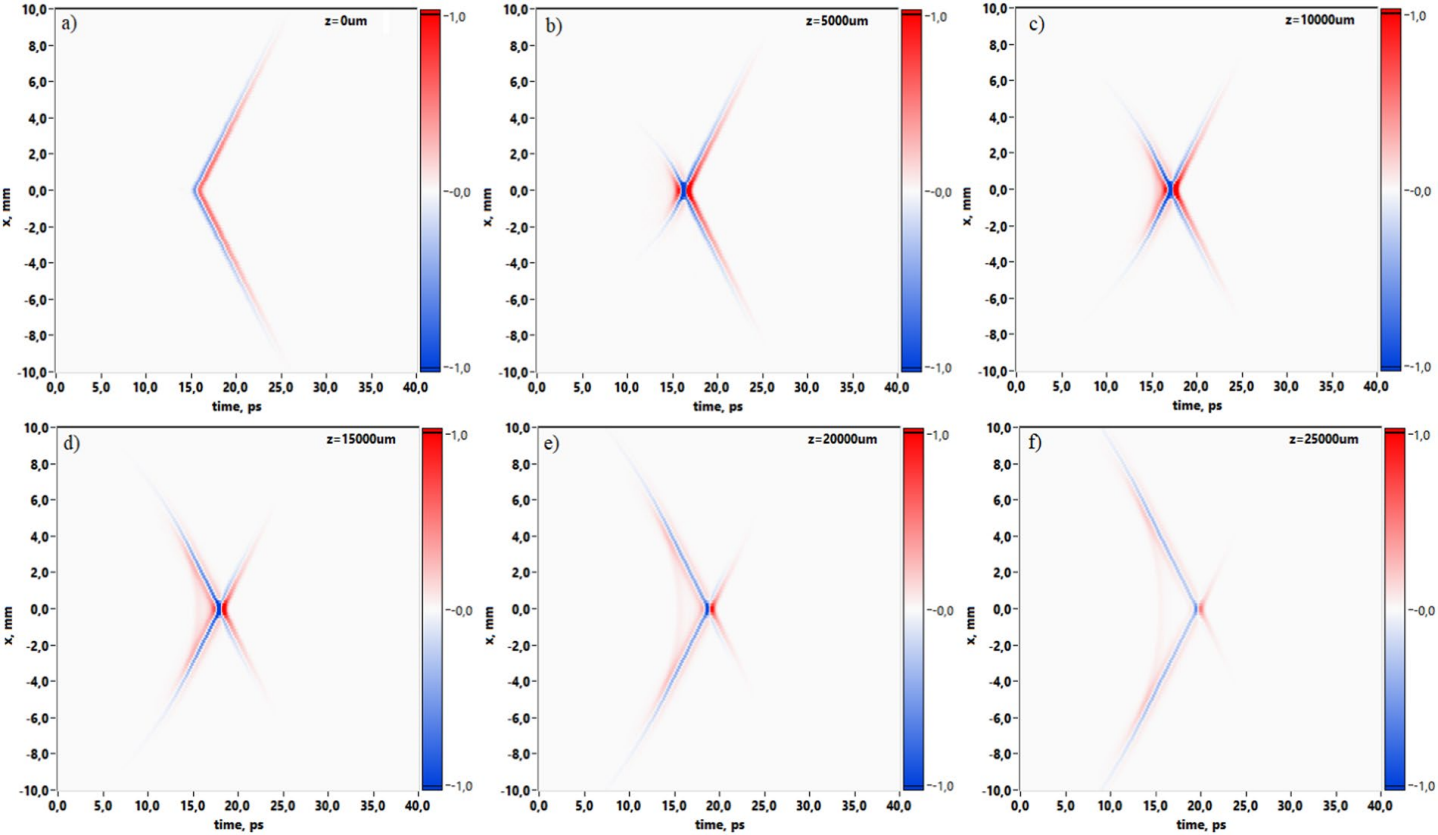
Spatio-temporal distributions of the THz field in the central cross-section of a Gauss-Bessel beam at different propagation distances: (**a**) corresponds to the initial wave front after axicon at *z* = 0 mm, (**b**) 5 mm, (**c**) 10 mm, (**d**) 15 mm, (**e**) 20 mm, (**f**) 25 mm.

The results are obtained with the use of THz PTDH technique described in detail in section “Methods”. The results shown in Fig. 1 illustrate the dynamics of Gauss-Bessel beam propagation in two-dimensional representation. Since the Gauss-Bessel beam is circularly symmetric, the spatio-temporal distribution in (*x*,*t*) plane provides comprehensive information about overall spatio-temporal beam properties at each coordinate of propagation axis. Temporal field evolution is depicted in coordinate system with time delay *τ* = *t* − *z*/*c*. The coordinate system moves at a constant group velocity taken equal to speed of light in vacuum *c* along the optical axis. This coordinate system minimizes temporal pulse shift and thus allows clear observation of pulse wavefront reshaping since for short pulses observations their shape changing is more important than its uniform propagation at a group velocity^38^. Figure 1 demonstrates the distribution of the THz field *E*(*t*, *x*) in the central cross-section of Gauss-Bessel beam via propagation distance in the range from 0 to 25 mm. In order to demonstrate the beam evolution, these temporal forms are normalized to global electric field amplitude maximum. Temporal wavefront evolution during its propagation along *z*-axis over the distance range from axicon plane to *z* = 25 mm (where the Bessel beam exists) are presented with small step increment Δ*z* = 100 *μ*m (see movie 1).

**Figure 2:**
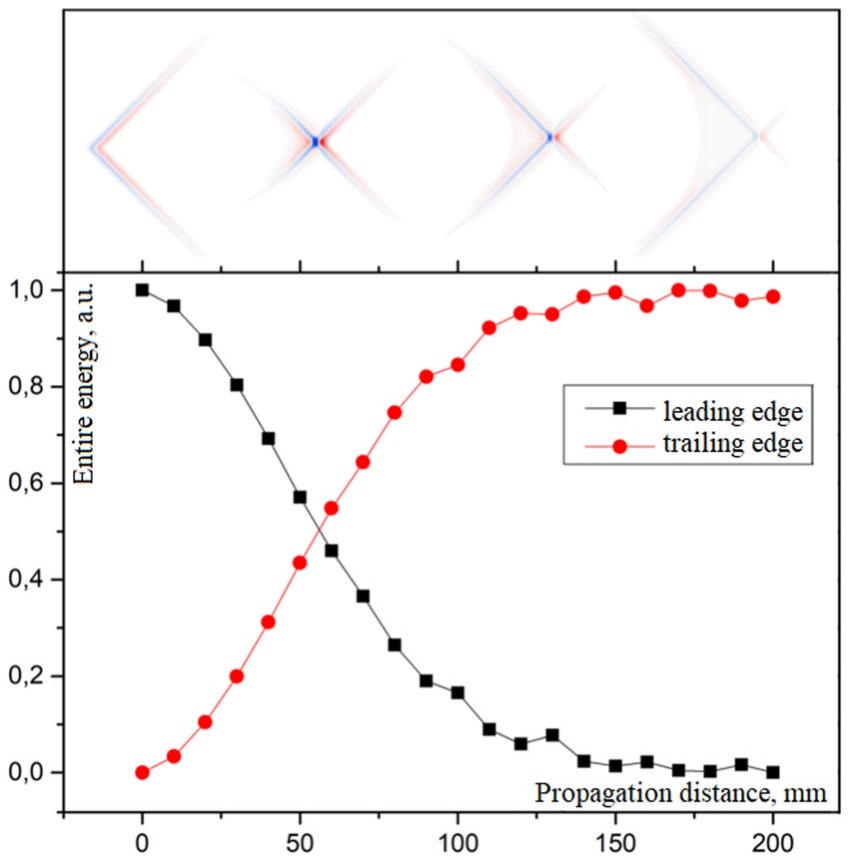
The dependency of pulse energy transfer via propagation distance.

2D field patterns in the Fig. 1 show the wavefront inversion as the propagation distance increases. Moreover, during the propagation strong pulse reshaping and X-shape structure formation occurs (Fig. 1c). This X-shape spatio-temporal distribution was investigated previously with the usage of optical 2f-2f imaging system, and the results of the study showed strong STC in the propagation of few-cycle pulses^27^. STC in this case was explained by the cutoff effect introduced by the focusing lens. Moreover, the cutoff effect appears strongly for few-cycle pulses in contrast to the long pulse, where its spatial distribution is time independent. Pulse reshaping in our research could be visually illustrated by energy transfer dependency via propagation, when energy flows from leading edge to trailing edge (see Fig. 2).

**Figure 3:**
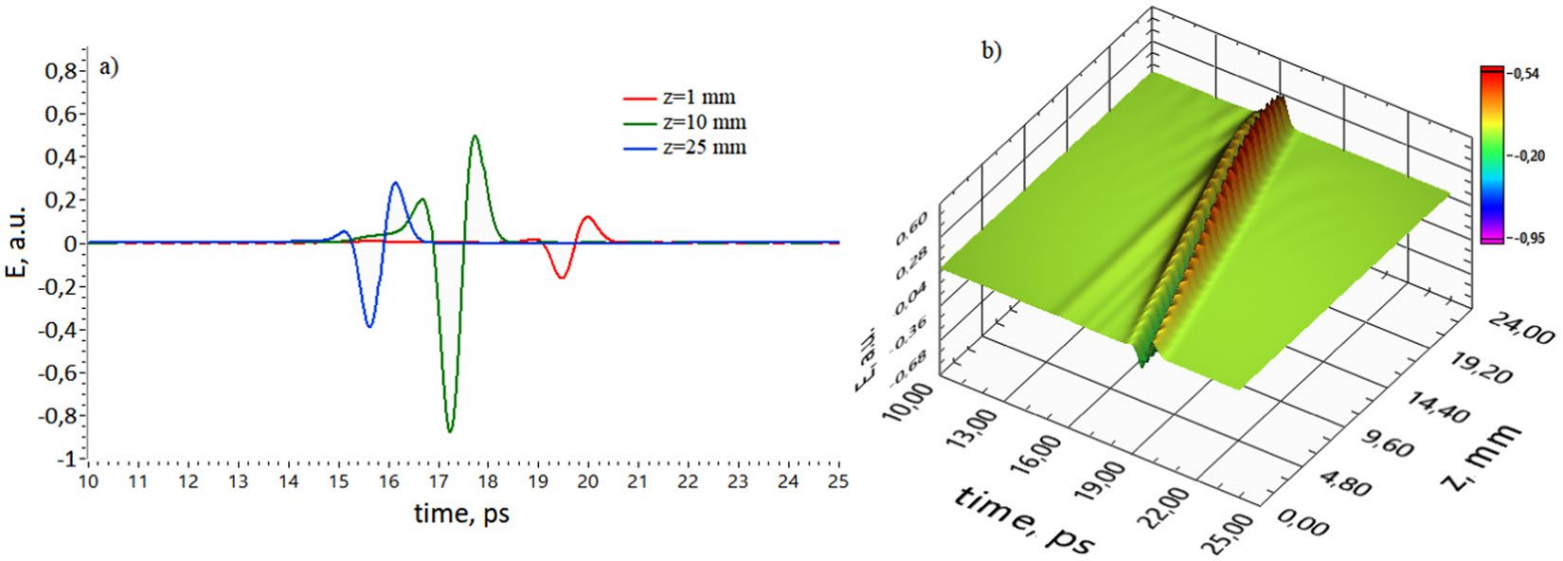
(**a**) 1D on-axis electric field distributions of the THz pulse at different propagation distances (blue line corresponds to *z* = 1 mm, green corresponds to *z* = 10 mm, red corresponds to *z* = 25 mm). (**b**) 3D view of on-axis THz pulse propagation.

Indeed, THz pulse is displaced at the temporal scale for ~5 ps (here we use running temporal axis). This is explained by the non-paraxial propagation of lateral wavefronts and their interference. More clearly it is shown in 1D and 3D representation in Fig. 3, which depicts THz pulse evolution along the optical *z*-axis.

### Spatio-spectral evolution

**Figure 4:**
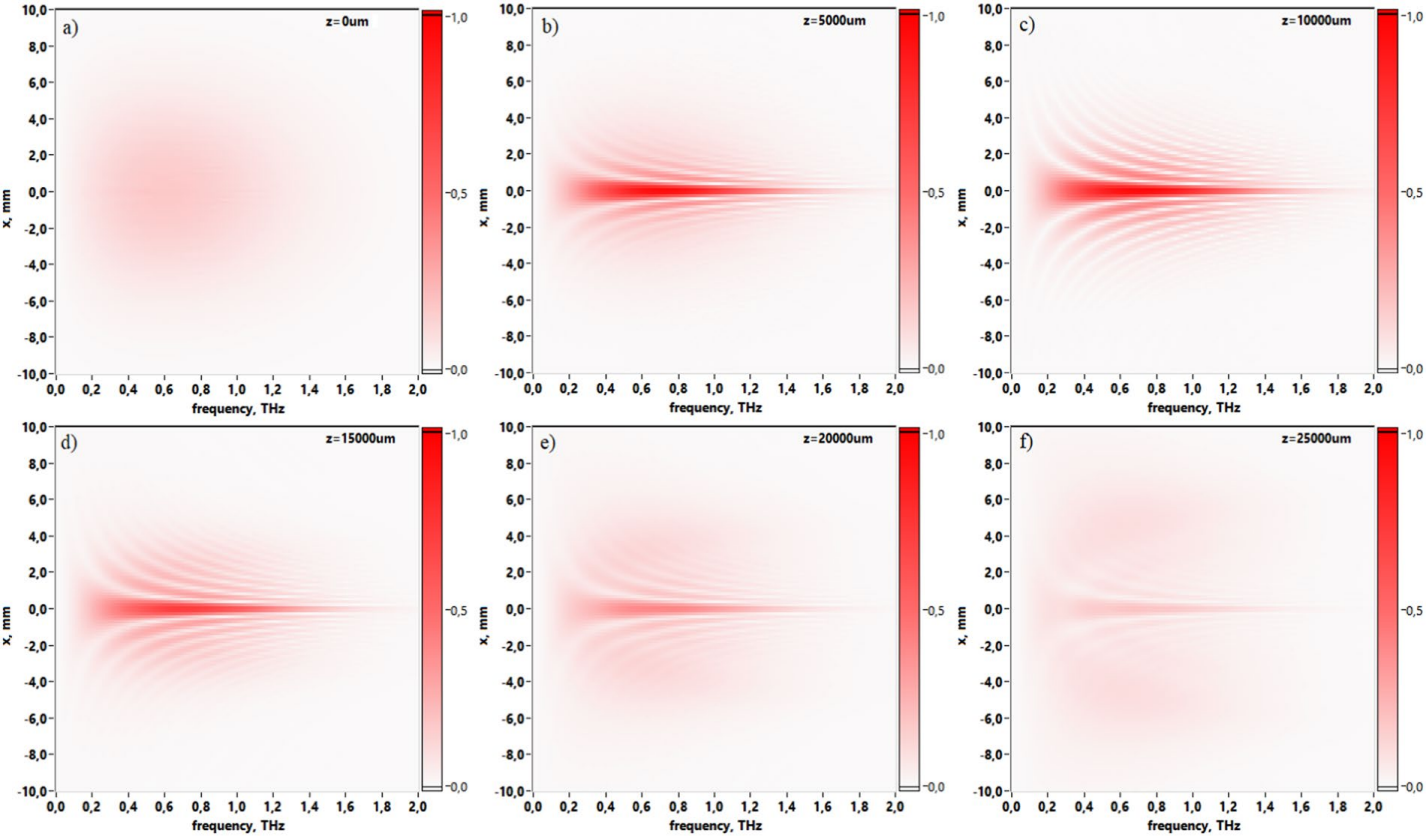
Spatio-spectral distributions of the THz field amplitude in the central cross-section of the Gauss-Bessel beam via propagation distance: (**a**) corresponds to the initial wave form when *z* = 0 mm, (**b**) 5 mm, (**c**) 10 mm, (**d**) 15 mm, (**e**) 20 mm, (**f**) 25 mm.

Due to the broadband nature of pulsed THz Gauss-Bessel beam it is useful to observe its spatio-spectral evolution via propagation distance. Figure 4 illustrations provide 2D dependencies of spectral amplitude in central cross-section of the Gauss-Bessel beam. The sequence of images illustrates changes in the spectral amplitude distribution for the corresponding distances from 0–25 mm with increment Δ*z* equal to 5 mm. In detail these 2D spectral amplitude profiles *U*(*x*, *ν*) consist of a bright central spot surrounded by weaker interference rings. This spatio-spectral pattern forms after axicon and changes during propagation. At distance *z* = 25 mm the central peak of spectral amplitude becomes equal to the amplitude distribution at the edge of the beam, thus illustrating that the beam is already disintegrated. Moreover, each pattern in Fig. 4 corresponds to the first-order Bessel functions which depend on THz frequencies. The spatio-spectral lateral decreasing behavior is attributed to the propagation of wavefronts at some angle to the optical axis and their mutual interference. This behavior could be refered to the frequency factor in wavefront propagation equations (see section Methods, eqs. (5), (7)). Analyzing the spectral evolution with small increment Δ*z* = 100 *μ*m (see movie 2) in region from axicon plane to *z* = 25 mm it becomes obvious that the Gauss-Bessel beam exists in this distance range.

**Figure 5:**
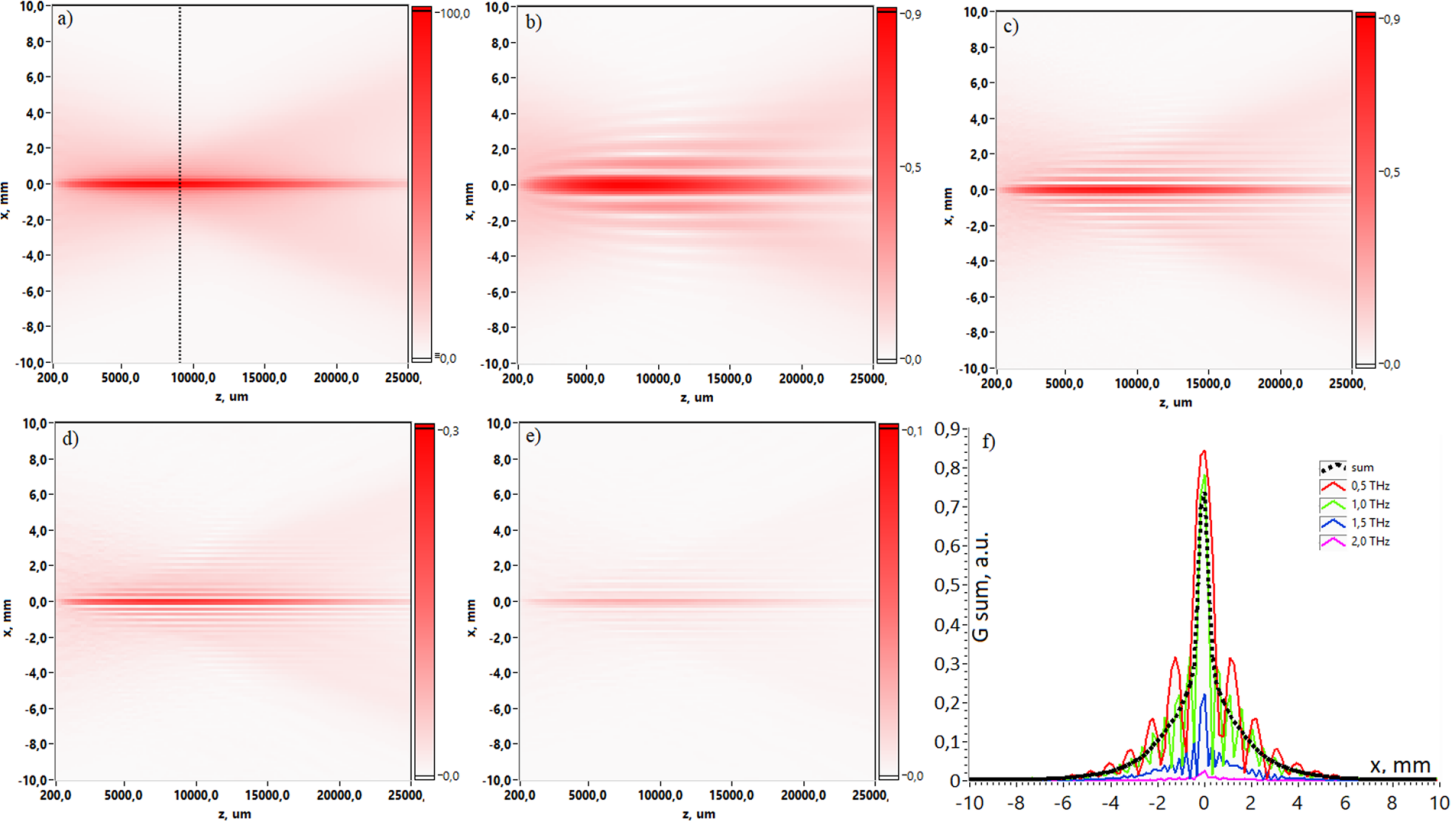
Longitudinal patterns of Gauss-Bessel beam’s amplitude distribution via propagation distance *z*. (**a**) corresponds to the broadband frequency range 0,05–2 THz, (**b**–**e**) correspond to frequencies *ν* = 0,5 THz, 1 THz, 1,5 THz and 2 THz consequently. (**f**) depicts cross-sections for fixed distance *z* = 9,5 mm. The cut place is indicated by dot line in the (**a**).

The longitudinal evolution is demonstrated in Fig. 5 for several monochromatic components as well as for summarized spectral range from 0,05 to 2 THz. Figure 5f provides detailed consideration showing cross-sections for fixed distance *z* = 9,5 mm (the cross-section is indicated by dot line in Fig. 5a). One can see that different frequency components provide different patterns of maximum and minimum of spectral amplitude due to the mutual interference of radially symmetric wave planes formed after axicon and then propagating at some angle to the optical axis. The cross-section corresponding to the broadband frequency range 0,05–2 THz illustrates spreading of lateral interference pattern for the fixed distance *z*. Thus, the frequency summarized structure in Fig. 5a shows the narrow Gauss-Bessel non-diffractive beam in the integral form without interference rings how it could be detected in the experiment.

### Phase velocity estimation

Lloyd *et al*.^26^. estimated the phase velocity of THz Gauss-Bessel beam experimentally. However, these values were measured only for paraxial case, i.e. on the optical axis in a single point. Moreover, for waves of a more complicated form in contradistinction to monochromatic plane waves the phase velocity differs in general from *c*/*n* and varies from point to point even in a homogenius medium^39^. Anyway, according to the theory of relativity, signals can never exceed *c*. This implies that the phase velocity cannot correspond to a signal propagation velocity.

**Figure 6:**
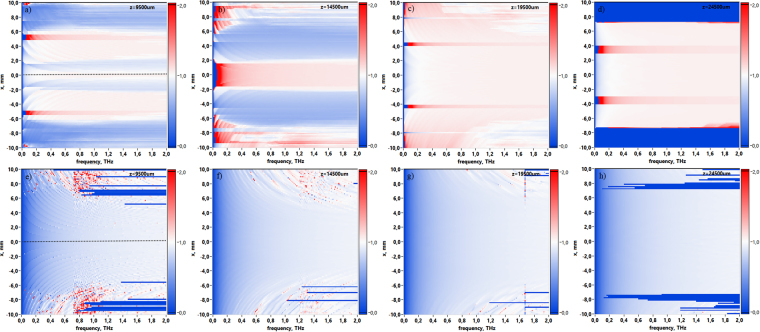
The phase velocity distribution via THz frequency for different distance increment. Upper row corresponds to Δ*z* = 5 mm, lower row corresponds to distance increment Δ*z* = 5 *μ*m.

Experimental measurements^26^ of the phase velocity was provided in case of THz waveforms with distance increment Δ*z* equal to 5 mm along *z*-axis. The phase velocity is calculated according to *V*_*ph*_ = 2*πν* ⋅ Δ*z*/Δ*φ*. Thus, calculation of the derivative value of a discrete function may play a substantial role. From a mathematical point of view, the derivative definition implies the calculation of the function change on ultra-small intervals. However, there are examples where, from certain physical considerations, the intervals of the argument over which the differentiation is performed are chosen to be sufficiently large, for instance, in the case of deterministic phase retrieval^40^. In work^26^, apparently, the calculation of the derivative over such a large interval was due to the peculiarity of the experimental scheme or the difficulty of the operational measurements. Thus, the convergence to the exact value of *V*_*ph*_ via grid pitch decreasing deserves additional approval. Due to the THz pulse duration of 2 ps (this is equal to 600 *μ*m in space), step size of 5 mm corresponding to ~10 longitudinal beam sizes may be too big for clear superluminal effect observation. In present research we show that with THz PTDH technique it is possible to monitor the evolution of the broadband THz field both at big and small distances. Here we simulated full spatial-temporal evolution of pulsed THz Gauss-Bessel beam with different distance increment values. Figure 6(a–d) demonstrates spatio-frequency distributions of the phase velocity *V*_*ph*_(*x*, *ν*) with distance increment 5 mm starting from *z* = 4,5 mm similar with paper^26^. For the estimation of the phase velocity behavior for the case of the small increment we simulated the beam propagation evolution using Δ*z* equal to 10 *μ*m (Fig. 6(e–h)).

**Figure 7:**
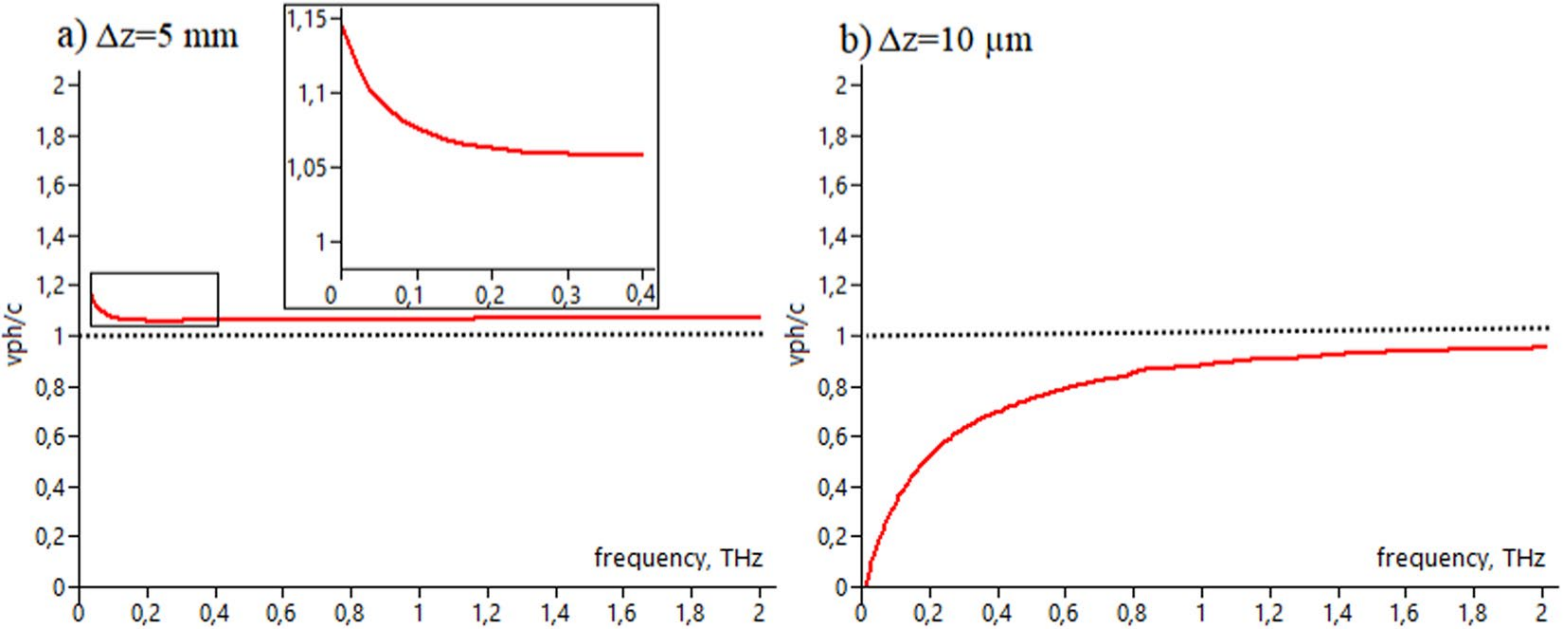
Central cross-section (the cut place is indicated by dot line in Fig. 6a,e) of the phase velocity distribution via THz frequency for different propagation distance increment. (**a**) corresponds to Δ*z* = 5 mm, (**b**) corresponds to Δ*z* = 5 *μ*m.

The experimental results in paper^26^ represented averages over several pairs of measurement in distance range from 4,5 mm to 24,5 mm with increment 5 mm. In order to compare these values Fig. 7 depicts averaged *V*_*ph*_ for central *x*-plane cross-section marked by dot line in Fig. 6a,e. It is clearly seen that for increment 5 mm *V*_*ph*_ exceeds *c*. The inset in the Fig. 7a demonstrates the spectral range similar to the results shown in Fig. 4 in paper^26^, showing the approximate agreement of the frequency dependent phase velocity value. In this case, the oscillations in the phase velocity graph in Fig. 4 in^26^ could be attributed to the experimental measurements peculiarities, since there are no objective reasons for their appearance, as follows from the calculated graphs. On the other hand, calculations with small distance increment Δ*z* = 10 *μ*m demonstrates that the phase velocity behavior does not display superluminal effect. Moreover, *V*_*ph*_ dynamic is subluminal and approximates asymptotically to *c* via frequency increasing. Noisy behavior of the phase velocity at the edges of *x*-plane is associated with pulse size and corresponding THz amplitude decreasing due to the Gauss-Bessel beam reshaping during the propagation.

## Discussion

This paper investiagates the evolution of the pulsed THz Gauss-Bessel beam in non-paraxial case in the spatio-temporal and spatio-spectral representations, thus demonstrating strong spatio-temporal coupling. We have demonstrated THz pulsed Gauss-Bessel beam’s wavefront propagation, including formation of the X-shape structure and subsequent wavefront changes to the opposite orientation relative to the original position behind the axicon at *z* = 0 mm. The evolution of the phase velocity in the two-dimensional representation is discussed, comparing with the experimental measurements in previous work^26^, where THz waveforms were acquired at distance increment equal to 5 mm along *z*-axis. This can be crucial due to the fact that THz pulse with 2 ps duration occupies 600 μm in space. Thus, the phase velocity estimation requires spectral phase information of two waveforms measured at two different locations on *z*-axis according to *V*_*ph*_ = 2*πν* ⋅ Δ*z*/Δ*φ*. Therefore, step size of 5 mm corresponding to ~10 longitudinal beam sizes may be incorrect from the mathematical point of view, since the phase velocity is calculated as the discrete derivative Δ*z*/Δ*φ* with the assumption that the Δ*z* tends to zero. This can be important especially for Gauss-Bessel beam, where plane wave components after axicon propagate at some angle to the optical axis, but waveforms measurements are provided only along *z*-axis. As derivative of a discrete function Δ*z*/Δ*φ* is more accurate when increment is smaller, the question whether it is possible to estimate the phase velocity using Δ*z* step about 10 pulse sizes needs an additional approval. Thus, convergence to an exact value as the grid step is reduced, is a significant aspect for superluminal observation. In our research we have shown that results of the phase velocity calculation depend strongly on propagation increment value, thus demonstrating superluminal (Fig. 7a) or subluminal behavior (Fig. 7b).

## Methods

**Figure 8:**
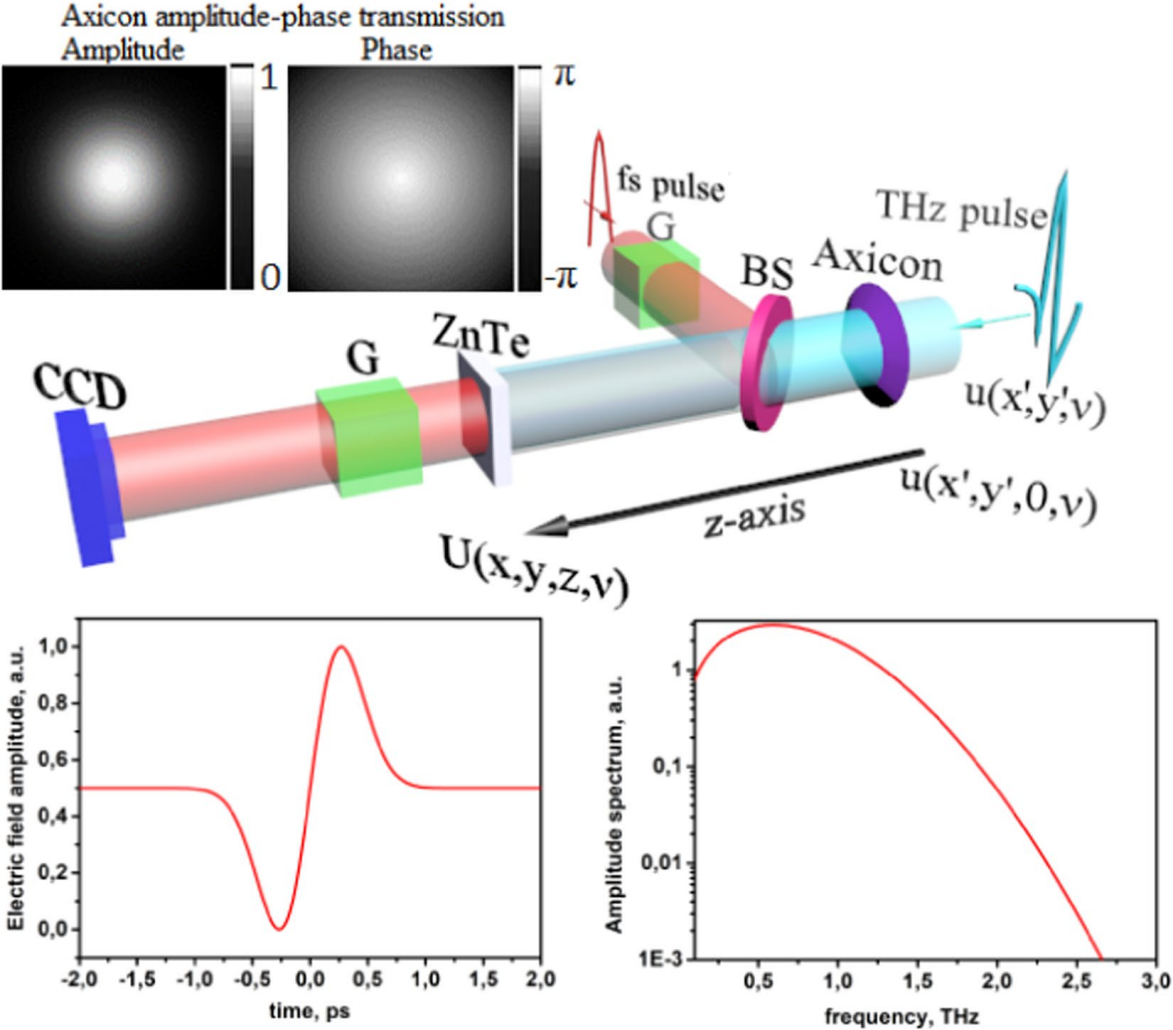
Scheme of the propagation of pulsed THz radiation through the phase axicon with the formation of a Gauss-Bessel beam. A collimated wide-aperture THz beam illuminates the phase axicon located at the distance *z* from the ZnTe detection crystal. A wide-aperture probe beam of pulsed femtosecond radiation is propagated to the detection crystal and further recorded by a CCD array. The polarization of this optical femtosecond probe beam changes under the influence of the applied external field from the THz beam due to the electrooptical effect in ZnTe crystal. Thus, the polarization of the femtosecond probe beam is proportional to the amplitude of the THz field, which is recorded by the CCD matrix as a change of input intensity. Glane prisms (G) control the change of the polarization state in the probe beam, since two polarizers are in a crossed state at 90 degrees. The temporal resolution of the THz signal is achieved by changing the time of the intersection of the probe and THz beams in the ZnTe crystal with the moving of the optical delay line. By changing the position of the axicon it is possible to obtain pictures of THz fields at different propagation distances of the Gauss-Bessel beam. This scheme allows determining the dynamics of THz Gauss-Bessel beam propagation and estimation the phase velocity behavior. In the numerical simulation of the propagation of pulsed THz Gauss-Bessel beam we used the following parameters: THz pulse duration parameter *τ* was 0.38 ps, the number of points in the temporal profile of the pulse was 2048, the time window size was 100 ps, the central frequency was 0.66 THz. For the phase axicon the maximum height was 9.33 mm, which provides the base angle of 43 degrees, transverse dimension of the axicon was 20 mm. This parameters correspond to the values, described in the experimental work^26^, what allows us to directly compare the results of our model experiment with the experimental data obtained earlier. The refractive index of the axicon material is 1.46, which corresponds to the Teflon usually used in experimental studies because of the high degree of transparency in the THz frequency range^36,43^.

The investigation scheme is depicted in Fig. 8. The wide-aperture THz beam propagates through the phase axicon, which is formed by the corresponding amplitude-phase transmission in the approximation of an infinitely thin phase object. According to works^41,42^ original THz pulse is a single-cycle pulse, whose electric field amplitude *E*(*t*) can be represented by the following function:

1 $$E(t)={E}_{0}\frac{t}{\tau }\exp (-\frac{{t}^{2}}{{\tau }^{2}}),$$

where *E*_0_ is the amplitude of electric field cycles, and *τ* sets the pulse duration value. The THz pulse, which is an ideal one-period structure with FWHM of the envelope of the modulus square of *E*(*t*) about 1 ps, decomposes through the Fourier spectrum into frequencies *ν*:

2 $$u(\nu )={ {\mathcal F} }_{1D}(E(t))={\int }_{0}^{\infty }E(t)\exp (-2\pi i\nu t)dt\mathrm{.}$$

These spectra are complex functions $u(x^{\prime} ,y^{\prime} ,\nu )=|u(x^{\prime} ,y^{\prime} ,\nu )|\exp (i\phi (x^{\prime} ,y^{\prime} ,\nu ))$ that contain both amplitude |*u*(*x*′, *y*′, *ν*)| and phase *φ*(*x*′, *y*′, *ν*) components in each (*x*′, *y*′) point at *z*-plane. Then for each spectral component the field *u*(*x*′, *y*′, *ν*) is layerwise multiplied on the amplitude transparency mask *T*(*x*′, *y*′, *ν*), thus forming initial 3D data array at *z* = 0 mm. Here coordinates (*x*′, *y*′) indicate at the initial plane where the axicon is placed:

3 $$u(x^{\prime} ,y^{\prime} \mathrm{,0,}\nu )=u(x^{\prime} ,y^{\prime} ,\nu )\cdot T(x^{\prime} ,y^{\prime} ,\nu )$$

Here transparency mask *T*(*x*′, *y*′, *ν*) corresponds to 2D Gaussian distribution (Fig. 8, inset), which characterizes amplitude of wide collimated THz beam. If the beam is ideally collimated, the dependence of field amplitude via frequency can be neglected. However, in practice, it may not occur if to use, for instance, chromatic focusing elements. In present calculation we use the approximation where the THz beam is collimated and is not affected by diffraction divergence during small propagation distances from the THz source to the axicon plane. The phase mask corresponding to the axicon relief *H*(*x*′, *y*′) is transformed into a phase distribution according to the equation:

4 $$\phi (x^{\prime} ,y^{\prime} \mathrm{,0,}\nu )=\frac{2\pi \nu }{c}(n(\nu )-\mathrm{1)}H(x^{\prime} ,y^{\prime} )$$

Here *H*(*x*′, *y*′) is axicon relief distribution, *n* is refractive index of the axicon’s material. Thus, a passage of the THz field through the axicon with relief *H*(*x*′, *y*′) is equivalent to the 3D data array of phase delay *φ*(*x*′, *y*′, *ν*) for each spectral component *ν*. It is also possible that the material from which the diffractive element is made has a dispersion. A detailed study on this issue is published in the paper^44^, but now this case is beyond the scope of our consideration. Diffraction by the axicon also can be described using Maxwell theory with the finite-element method^45^.

Here we use a scalar theory of diffraction realized by THz PTDH^36^. The resulting two-dimensional complex wave field $u(x^{\prime} ,\,y^{\prime} ,\,\mathrm{0,}\,\nu )=|u(x^{\prime} ,\,y^{\prime} ,\,\mathrm{0,}\,\nu )|\exp (i\phi (x^{\prime} ,\,y^{\prime} ,\,\mathrm{0,}\,\nu ))$ after the axicon propagates along *z*-axis. Wavefront propagation from object plane *u*(*x*′, *y*′, 0, *ν*) to arbitrary plane *U*(*x*, *y*, *z*, *ν*) is realized by the angular spectrum (AS) and Rayleigh-Sommerfeld convolution (RSC) methods^46^. AS method for wavefront numerical propagation is defined as follows:

5 $$\begin{array}{c}U(x,y,z,\nu )=\underset{-{\rm{\infty }}}{\overset{+{\rm{\infty }}}{\iint }}u({f}_{x},{f}_{y})\exp (2\pi i(x{f}_{x}+y{f}_{y}))\exp (-i2\pi z\sqrt{\frac{{\nu }^{2}}{{c}^{2}}-({{f}_{x}}^{2}+{{f}_{y}}^{2})})d{f}_{x}d{f}_{y},\end{array}$$

where

6 $$\begin{array}{c}u({f}_{x},{f}_{y})=\underset{-{\rm{\infty }}}{\overset{+{\rm{\infty }}}{\iint }}u(x^{\prime} ,y^{\prime} ,0,\nu )\exp (-i2\pi (x^{\prime} {f}_{x}+y^{\prime} {f}_{y}))dx^{\prime} dy^{\prime} \end{array}$$

is the angular spectrum represented through the spatial frequencies (*f*_*x*_, *f*_*y*_).

Similarly, the field *U*(*x*, *y*, *z*, *ν*) can be calculated using the Rayleigh-Sommerfeld convolution (RSC) method:

7 $$U(x,y,z,\nu )=u(x^{\prime} ,y^{\prime} ,0,\nu )\ast h(x,y,z,\nu )$$

where *h* is the pulse response function

8 $$h(x,y,z,\nu )=\frac{\nu \,\exp (i2\pi r\nu {c}^{-1})}{i\cdot c\cdot r}\frac{z}{r}$$

and *r* is the distance between the object and registration planes:

9 $$r=\sqrt{({z}^{2}+{(x-x^{\prime} )}^{2}+{(y-y^{\prime} )}^{2})}$$

In case of broadband THz radiation, the choice of the calculation method is determined by the critical frequency *ν*_0_, depending on the distance *z*, the grid size *N*, the grid pitch Δ*x* which should be equal to Δ*y* (see the paper^47^) and the refractive index dispersion *n*(*ν*):

10 $${\nu }_{0}=cz/n(\nu )N{\rm{\Delta }}{x}^{2}$$

Thus, AS method is used when $\nu \ge {\nu }_{0}$, and RSC is used otherwise for *ν* < *ν*_0_. There are some experimental techniques providing temporal forms of THz electric field. In particular the method of electrooptical detection is illustrated in Fig. 8, where cross-polarization scheme is used^48,49^. In this configuration the registration plane represents the plane where THz detection crystal ZnTe is located, and the plane of the object is the axicon itself (see Fig. 8). Therefore, it is possible to vary the propagation distance Δ*z* by axial displacement of the phase axicon from the detection crystal according to the assumpion that the incident THz beam with Gauss profile do not change significantly its form via axicon displacement. The main feature of the THz holography scheme is the possibility of measurement of the wide aperture collimated THz beam and the recording of THz electric field in time-domain. This method^36^ allows numerical investigation of the dynamics of wave field in temporal *E*(*x*, *y*, *z*, *t*) and spectral *U*(*x*, *y*, *z*, *ν*) representation in arbitrary plane. Thus, one can reconstruct the complex amplitude-phase characteristics of radiation in an arbitrary located plane by solutions of numerical wavefront propagation equations (5) and (7). This experimental possibility allows the simulation in approximation of wide collimated pulsed THz beam.

THz PTDH technique provides a possibility to estimate the evolution of electric field of beams by estimating and measuring their propagation dynamics in the representation of 5D-data: three-dimensional spatial coordinates, temporal and spectral scales, including amplitude and phase information in the complex spectrum data. This method is a powerful tool of obtaining an amplitude-phase THz fields with high resolution, which allows to display the spectroscopic information of the object under study. It also allows the accounting of optical parameters, such as a refractive index dispersion of the object material and propagation media. The method demonstrates the ability to reconstruct smooth and stepped relief objects or an object that is transparent in the THz region, as well as to estimate the complex spatial-temporal evolution of the complex THz field. Theoretical plausibility and repeatability of THz PTDH is confirmed in a variety of experimental and simulation works^36,37,44,46,50–52^.

## Conclusion

THz PTDH method could be applied for arbitrary beams which can be formalized by initial amplitude-phase spatial distribution. In this case THz PTDH approach can perform simulation of wide aperture beams, thus expanding numerical solutions of wavefront propagation to non-paraxial case. This is especially actual for the beams which propagates at some angle to the optical axis, for example Gauss-Bessel beams, formed by axicon with high base angle. Reliability of THz PTDH was proved including by comparison of simulated wavefront with the wave field experimentally obtained after its passing through an arbitrary phase object (see Fig. 5 in paper^36^). In present research we observed the evolution of THz pused broadband Gauss-Bessel beam in spatio-temporal and spatio-spectral representation, demonstrating all stages of pulse reshaping during propagation, including X-shape pulse forming and analyzing the energy transfer dynamics, where pulse energy flows from leading edge to trailing edge. THz PTDH also provided the illustration of STC effect of Bessel beam’s wavefront propagation. The beam’s profile structure in the temporal and spectral domain clearly illustrated the model when the wave front of the Gauss-Bessel beam at the exit from the axicon can be divided into radial segments for which the wave vectors intersect during propagation. The phase velocity via propagation distance was estimated and compared with existing experimantal results^26^. The superluminal or subluminal behavior depends strongly on distance increment value in compariosn with the size occupied by the THz Gauss-Bessel pulse in space: for the high value of Δ*z* like in^26^ superluminal effect is observed and for the small Δ*z* subluminal effect is observed.

## Electronic supplementary material

movie 1

movie 2
